# Opioid Consumption in Chronic Pain Patients: Role of Perceived Injustice and Other Psychological and Socioeconomic Factors

**DOI:** 10.3390/jcm11030647

**Published:** 2022-01-27

**Authors:** Barbara Kleinmann, Tilman Wolter

**Affiliations:** Interdisciplinary Pain Center, Faculty of Medicine, University of Freiburg, 79106 Freiburg, Germany; barbara.kleinmann@uniklinik-freiburg.de

**Keywords:** chronic pain, perceived injustice, opioid use, socioeconomic factors, psychological factors, lifestyle

## Abstract

Background: Chronic pain is a complex biopsychosocial phenomenon. Lifestyle, behavioral, socioeconomic, and psychosocial factors such as depression and perceived injustice are often associated with the development of chronic pain and vice versa. We sought to examine the interaction of these factors with opioid intake. Methods: At our institution, 164 patients with chronic pain undergoing an interdisciplinary assessment within a three-month period participated in the study and completed the Injustice Experience Questionnaire (IEQ). Data regarding opioid intake, pain levels, pain diagnosis, depression, anxiety, stress, quality of life, pain-related disability, habitual well-being, occupational status, and ongoing workers compensation litigation were extracted from the patients’ charts. Results: Approximately one-fourth of the patients used opioids. The IEQ total was significantly higher in patients using Schedule III opioids. Depression, but not the anxiety and stress scores, were significantly higher in patients using opioids. There were no significant differences regarding pain-related disability, habitual well-being, and the coded psychosocial diagnoses. In the patient group without opioids, the percentage of employed persons was significantly higher but there were no significant differences regarding work leave, pension application, or professional education. Conclusions: Opioid use appears to be more closely related to psychological factors and single social determinants of pain than to somatic factors.

## 1. Introduction

Chronic pain affects many aspects of daily activities, physical and mental health, family, social relationships, and workplace interactions [1]. In turn, all of these factors can also influence the perception of chronic pain [2]. Opioids can be an important tool in the management of chronic pain. However, the experience of recent years has shown that benefit and harm in treatment of non-cancer pain can be closely related, and that opioid consumption is influenced by different factors [3,4,5].

Studies on opioid prescriptions show that besides compromised lifestyle factors such as physical activity and functioning, psychological and socioeconomic factors such as work force participation and social capital contribute to the amount of opioid consumption and the number of opioid-related deaths [1,6,7,8]. Opioid use is associated with statistically significant but small improvements in pain and physical functioning [9]. Numerous studies exist demonstrating that psychological comorbidities such as depression and anxiety are prevalent among patients with chronic non-cancer pain [3,6], and that these patients are more likely to receive long-term opioid therapy for pain [4]. A proposed reason for this phenomenon is that mental health conditions and chronic non-cancer pain are closely correlated concerning severity [5]. Moreover, patients with psychological comorbidities have a tendency to use opioids earlier and to use higher dosages of opioids [6], and opioid use may be a contributing factor for the development of depression [2].

Perceived injustice is a novel psychological variable interacting with chronic pain and opioid use. Scott et al. and Sullivan et al. showed that high levels of perceived injustice as measured with the Injustice Experience Questionnaire (IEQ) may also increase pain severity and depressive symptoms [10,11]. Sullivan et al. showed that high scores on perceived injustice are correlated negatively with recovery from mental health problems, poor rehabilitation outcomes, and prolonged work disability, and that the IEQ could possibly be used as a prognostic factor in the treatment of patients with chronic pain [12]. High scores on perceived injustice also predicted work disability, even if the initial pain intensity, functional limitations after the injury, catastrophizing, depression, and pain-related fears are controlled. Perceived injustice was more related to disability than to pain severity and it was the best predictor for occupational disability. Interestingly, catastrophizing was the best predictor for pain severity. Sullivan et al. suggested that perceived injustice should be further investigated in terms of its prognostic value for recovery [13].

Carriere et al. reported a correlation between perceived injustice and opioid prescription in patients with chronic pain [14]. They found that pain behavior, rather than pain intensity and depressive symptoms, mediated the association between perceived injustice and opioid prescription in patients with chronic pain. They discussed perceived injustice as a risk factor for adverse pain-related outcomes [14] and recommended future research in this area in order to identify more details and factors influencing the relationship of perceived injustice and opioid prescription. Moreover, Nijs et al. recently proposed that the assessment of perceived injustice, by means of the IEQ, should be included in the screening of cancer survivors with chronic pain because of its potential relevance for different treatment strategies including opioid medication [15]. While the correlation between depression, perceived injustice, and opioid use in chronic pain is well established, there is little knowledge about the possibly contributing socioeconomic factors. High perceived stress, e.g., due to high job demands and low control of decisions at work, was associated with more neck pain and decreased work productivity [16,17,18,19]. Occupational factors can also have a significant influence on the development of low back pain disorders [20]. Recently, Serra-Pujadas et al. [21] showed that socioeconomic status has a major influence on opioid use but their study was based only on regional insurance data.

The aim of this prospective study was to evaluate a possible correlation of opioid therapy in particular with socioeconomic factors and psychological factors such as the feeling of perceived injustice. For this purpose, we examined a representative group of patients with chronic non-cancer pain in a tertiary pain center.

## 2. Material and Methods

### 2.1. Patients

Inclusion criteria were: appointment in our institution for an interdisciplinary assessment between 1 October 2020 and 31 December 2020, age above 18 years, ability to understand and fill in the study questionnaires. Patients are treated in this department on an outpatient, inpatient and inpatient day-care basis. Prior to first presentation, patients routinely fill out the German Pain Questionnaire before then being admitted to our institution [22]. Assessment examinations are only given to patients who, based on the evaluation of the German Pain Questionnaire and the available medical findings, suffer from chronic pain with psychosocial stress factors and who have already undergone multiple frustrating pain therapies. This assessment is carried out in one day, i.e., the patient is inpatient for one day and is being looked after by an interprofessional team of doctors, physiotherapists, and psychologists during this time. [23]. Specialists from each discipline examine the patients for the causes of their chronic pain and the contributing chronification factors with the aim of appropriate, generally multimodal treatment [24].

Exclusion criteria were: insufficiently completed questionnaires, acute pain syndromes.

Of the 191 patients initially fulfilling the inclusion criteria, 164 gave written content to participate in this cross-sectional study. The IEQ (Injustice Experience Questionnaire, German version) was distributed to the patients, during their stay for the assessment [25].

### 2.2. Questionnaires and Data Extraction

The IEQ examines perceived injustice (sense of unfairness, severity of loss) as a contributing factor for the development of chronic pain [12,26]. The IEQ consists of 12 items with a 5-point scale (0–4), so that a maximal 48 points can be reached in total. Six items each form the subscale blame and the subscale severity. The cut-off value for the IEQ total score is 30; 14 for the subscale blame and 16 for the subscale severity [13,27]. The IEQ total score and the scores for the subscales blame and severity were calculated from the IEQ [13].

The German Pain Questionnaire was developed and validated by the German Chapter of the International Association for the Study of Pain (DGSS) [22,28]. The concept of this questionnaire is based on a bio-(medical)-psycho-social pain model. This questionnaire generates pain ratings on the 11-point numerical rating scale (NRS) and anxiety/depression/stress scores as measured by the German version of the Depression Anxiety Stress Scale (DASS) [29]. Patients rate their current, mean, maximum pain in the last four weeks and their bearable pain in case of successful pain treatment. The DASS consists of seven items each for depression, anxiety, and stress. In each of these items, 0–3 points can be reached. Values above 10 indicate an increased probability of the presence of chronic stress or a depressive disorder, while values above 6 are suspicious for anxiety. Moreover, for the experience of impairment, the German Pain Questionnaire contains a disability score, a shortened version of the Pain Disability Index (PDI) in which scale items are rated on an 11-point scale ranging from 0–10 [30]. The mean value of these 3 items multiplied by 10 gives the value for the disability score. The German Pain Questionnaire further includes the Marburg Questionnaire on Habitual Health Findings (FW 7), a 7-item questionnaire with a 6-point scale for each item [31].

Data on employment status, current sick leave, pension application, education, and marital status were also collected from the German Pain Questionnaire. Furthermore, personal data, medication, as well as coded diagnoses were extracted from the charts. Moreover, diagnoses based on the ICD-10 (International Classification of Diseases) [32,33] were derived from the patients’ charts.

The study was approved by the local Ethics Committee (IRB number: 20-1061). The datasets generated and/or analyzed during the current study are available from the corresponding author on reasonable request. The analysis of the contributing factors to the IEQ will be published separately.

### 2.3. Coded Diagnoses

For the analysis, pain diagnoses were further grouped by body region in the following categories: headache, facial pain, neck pain, low back pain, neuropathic pain, and widespread pain. Psychological diagnoses were grouped in the following categories: Chronic Pain Disorder with Somatic and Psychological Factors (ICD-10: F45.41) [34], depression, anxiety, sleep disorder. Psychosocial factors are coded under Z-diagnoses (factors influencing health status and contact with health services). These diagnoses were grouped in four categories: family (Z63), work (Z56), biography (Z61), and finance (Z59). For instance, Z-diagnoses pertaining to the family are coded in case of severe conflicts within the family. Work factors are coded in case of imminent loss of employment or severe conflicts in the working environment. Biographical Z-diagnoses are coded in case of childhood trauma, parental neglect, or in some cases loss of parents during childhood, while financial Z-diagnoses are coded in case of severe financial problems, i.e., massive debts or imminent loss of housing.

### 2.4. Statistical Analysis

A computer software package (GraphPad Prism, Version 5.01, GraphPad Software, Inc., La Jolla, CA, USA) was used to conduct statistical analyses other than the regression analysis, which was performed with SPSS (IBM SPSS Statistics for Windows, Version 27.0, Armonk, NY, USA). Initially, descriptive statistics were applied to all measures. An unpaired t-test (in case of normally distributed variables) and, in the more frequent case of missing Gaussian distribution, the Mann–Whitney Test were used to determine the statistical significance of the differences in mean scores. Comparisons with categorical variables were made by means of the chi-squared test and, if indicated, Fisher’s exact test. Statistical significance was considered when *p* < 0.05. The sample size estimation was performed with G*Power [35]. The sample size was 164 for the Mann–Whitney Test with α = 0.05 and a power of 0.8 and an effect size of 0.4. Logistic regression analysis was used to investigate the relation between the variables found significant in the individual comparisons between patients with and without opioid use (plus age and sex).

## 3. Results

### 3.1. Patients

**Figure 1:**
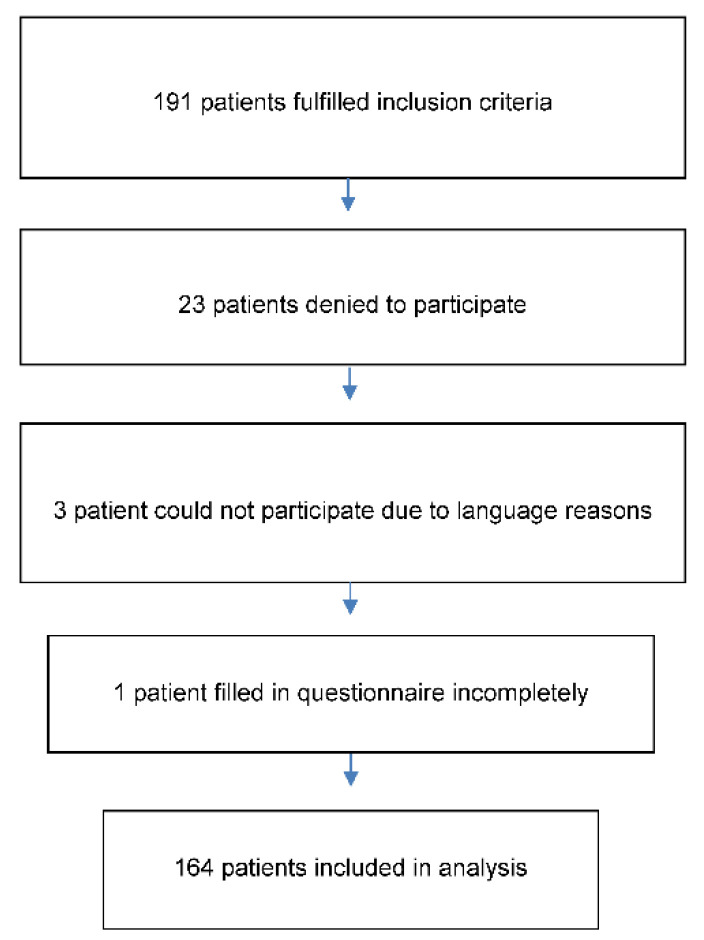
Flowsheet of patients eligible and patients analyzed.

Of the 191 patients initially fulfilling the inclusion criteria, 164 were included in the analysis (Figure 1).

Mean age was 50.3 years and nearly two-thirds of the patients included were female. Among the pain localizations, lumbar pain (low back pain) was most frequent followed by head and face pain, cervical pain, and widespread pain.

The median total pain score was 7.33 (IQR: 6.33–8.0). Almost 25% of the patients used opioids (39/164) equally divided between Schedule II and Schedule III opioids. Most of the patients (59.1%) used non-opioids or a single compound (55.5%). The proportion of patients who took anticonvulsants (18.3%) and antidepressants (21.9%) was roughly evenly distributed (Table 1).

The median scores for depression, anxiety, and stress within the study population were below the cutoffs for conspicuous or probable disorder. With regard to education, marital, and professional status, the following results were obtained: More than half of the patients were employed, one-third of the patients were unemployed or retired, and the rest of the patients received a disability pension. Most of the patients had no pension application, while 12 patients had. A non-academic professional education was reported by 78.05% of the patients (128/164). Two-thirds of the patients were married (Table 1).

### 3.2. Opioid Use, Gender, Age, Pain Localization, and Pain Diagnosis

No statistically significant differences were found among the proportion of opioid users between male and female patients. Moreover, there were no differences in age between patients with and without opioid use (Table 2).

There were no statistically significant differences found in pain localizations among patients with and without opioid use (Table 2).

Mean pain scores were higher in the group of patients taking opioids compared to those without opioid therapy. No statistically significant correlations between the other pain scores were found (Table 3).

### 3.3. Opioid Use and Psychological Factors

The IEQ total, but not the subscales blame and severity, was significantly higher in patients using Schedule III opioids than in those using no opioids. Considering all opioids (Schedule II and Schedule III opioids), this difference was no longer statistically significant. This was the only item which yielded different significance in patients taking Schedule III opioids than in patients taking Schedule II or III opioids, or both. The DASS depression and the DASS total score, but not the DASS anxiety and stress scores, were significantly higher in patients with opioid therapy compared to patients with no opioid therapy. There were no differences regarding pain-related disability and habitual well-being (Table 4), and no statistically significant differences in the frequency of coding of diagnoses such as “Pain Disorder with Somatic and Psychological Factors” (ICD-10: F45.41) [34], depression, anxiety, or sleep disorder (Table 5).

### 3.4. Opioid Use and Social Factors

There were significant differences in the occupational status between the patient groups with and without opioids. Logistic regression analysis showed that occupational status had a high correlation to opioid use. The overall model was significant, *p* < 0.001 (Table 6). No differences were found in the incidence of work leave or pension application or with different educational levels. Among the coded psychosocial diagnoses, there were no statistically significant differences between the patient groups with and without opioids (Table 5).

## 4. Discussion

In this prospective study, 24% of all investigated patients with chronic pain consumed opioids. There was no significant correlation between age, gender, and opioid consumption (Table 2). In contrast, other studies on the subject of gender-specific differences in patients with chronic pain found that women suffer from pain more often and also report higher pain intensity and more pain problems. This led to the conclusion that women were prescribed more opioids than men [36,37]. In our study, there were also no statistically significant age-related differences in opioid consumption behavior. However, a national population-based survey by Hudson et al. found that individuals older than 60 years were less likely to receive opioids than younger individuals [38].

Also pain localization showed no differences in the frequency of opioid consumption. In our study the majority complained of lumbar back pain (Table 3). In agreement with our study result, lumbar back pain is one of the most complained of pain syndromes in the western countries, with a global point prevalence estimated to be 9.4% [39]. We found no other references examining the relationship between different pain localizations and opioid consumption.

Opioid consumption was not related to most of the pain scores (Table 4), but interestingly, only mean pain was significantly higher in the group of patients taking opioids than in those without opioids. This result could confirm previous study results which report that opioid users were more likely than non-users to report high levels of pain interference with their daily lives [38]. On the other hand, Chen et al. reported on the lack of connection between the opioid dose change (increase or decrease) and the clinical pain score in a group of patients with chronic pain, regardless of age or gender [40]. These results were confirmed in further studies. Escalation of opioid dose was either not associated with improvements in NRS pain scores or with mild but clinically insignificant improvements [41].

In contrast to other study data, our study results show no difference in the mean values of the habitual well-being or the disability score of patients taking opioids and those not taking opioids [42]. This could possibly be a dose-dependent or habituation effect. Possible underlying mechanisms of a loss of efficacy of opioids in the sense of developing tolerance remain elusive, despite intensive research to understand the phenomenon [43]. Opioids may impair the assessment of one’s own quality of life through central nervous system side effects depending on the dose, speed of dose escalation and on comorbidities and co-medication [44]. Patients’ self-reported physical and psychological effects of opioid use in chronic non-cancer pain showed that improvement in general well-being irrespective of pain relief was experienced by 40% of the patients with chronic pain and opioid intake [45].

Wakaizumi et al. compared psychosocial, functional, and psychological measures between patients with chronic back pain who were managing their pain with or without opioids. Patients on opioids displayed poorer physical function [46]. In this context, it is important to know that our own non-pharmacological measures to improve the pain consist of self-reliant health attitudes and physical activities. Self-reliant health attitude, exercise, and physical activity have been shown to be a successful tool in avoiding opioids or discontinuing opioid use [47]. Further, a systematic review on opioids in patients with chronic non-cancer pain found small improvements in social functioning which were, however, far below the minimally important difference, and no improvements in emotional or role functioning [9].

The coded ICD-10 diagnoses of our study population, such as chronic pain disorder, depression, anxiety, and sleep disorder, had no significant correlation with opioid consumption. It is theorized that this could be the consequence of a relatively unspecific coding or diagnosis. An electronic health record such as the International Statistical Classification of Diseases and Related Health Problems, 10th Revision, German Modification (ICD-10-GM) is the official classification for coding diagnoses in outpatient and inpatient care in Germany. ICD-10 may receive insufficient underdiagnosis or outdated data if it is not updated regularly.

Depression showed a significant dependency on opioid intake in contrast to anxiety and stress. This result is partially consistent with Jamison et al., who reported that 40% of chronic pain patients treated with opioids suffer from additional affective disorders (depression and anxiety), which in turn are associated with a significantly increased misuse of opioids [36]. There are a number of studies demonstrating that people with psychological comorbidities such as depression and anxiety are prevalent among patients with chronic non-cancer pain [6,48,49,50], and that they are more likely to receive long-term opioid therapy for non-cancer pain than those without such comorbidities [4]. One reason for that could be that mental health conditions and chronic non-cancer pain are closely correlated concerning severity [5]. Moreover, patients with psychological comorbidities have a tendency to use opioids earlier and to use higher dosages of opioids [6]. Opioid use may be a factor for the new onset of depression, although the risk of depression is associated with longer duration of use but not with dose [2].

Consistent with previous research by Carriere et al. [14] we found a significant correlation between opioid consumption and perceived injustice (IEQ total) in our study population. Interestingly, this relationship was only confirmed for Schedule III opioids. If one assumes that patients with severe pain also prefer Schedule III opioids, this fits well with the results published by Carriere et al. This study group discussed that perceived injustice might contribute to higher levels of pain and as a consequence might increase the likelihood of opioid prescription [14]. For Carriere et al., pain behavior plays an important role in mediating between perceived injustice and opioid prescription. In a longitudinal study, Dickman et al. found that perceived injustice predicted increases in reported opioid use over three months, at least in patients without a high score in the PMQ (pain medicine questionnaire), thus in patients who did not take many other analgesics [51].

In our opinion, a therapeutic consequence for the reduction of opioids could be that patients should be screened for perceived injustice and receive psychoeducation or be counselled on that subject as appropriate. Other studies show that perceived injustice is a pain-influencing factor even in cancer survivors. Therefore, such patients should also be screened for perceived injustice as a trigger for behavioral patterns associated with opioid use [15]. Scott et al. even showed that perceived injustice augments the relationship between pain severity and depressive symptoms [10]. Based on the well-known relationships between depression and opioid consumption, one could argue that this observation could also be a cause of changed opioid consumption behavior.

As we already mentioned, there is a strong relationship between emotional stress and chronic pain. Furthermore, physical pain and negative emotions reinforce each other. This correlation is also shown in the fact that physical pain and negative emotions activate the same areas of the brain [52]. Opioids could be one way to treat not only physical pain but also social stress, and this could be a reason for the development of opioid abuse. Mark D. Sullivan emphasizes that “long-term opioid therapy impairs human social and emotional functions” [8]. Pain-related distress has been shown to increase pain intensity and interference [53,54,55] and to be associated with worse outcomes in treatment studies [56].

Concerning socioeconomic factors, only occupational status showed a significant correlation to opioid consumption. In addition, the logistic regression analysis showed that among the variables examined, occupational status had the strongest correlation with opioid use.

Employment status, education level, income, and occupational factors have already been discussed as risk factors for chronic pain [52,57]. To our knowledge, there are no proven correlations between opioid consumption and occupational status up to now. However, if one assumes that psychosocial stress, e.g., professional problems or problems in the workplace, is a risk for chronicity, and one knows that psychological stress can be associated with higher pain perception, a correlation between occupation and opioid consumption would be possible [57].

### Limitations

One limitation of this study could be that we do not know for sure whether there has been a change of medication or dosage between data collection from the German Pain Questionnaire and the IEQ. Since the time between data collection was only few weeks, clinical experience indicates that a substantial change is unlikely. Further, it should be taken into account that the socioeconomic data were submitted subjectively by the patients, e.g., patients may have classified themselves as incapacitated without stating whether this is an official assessment or an estimation. A type 2 error cannot be ruled out completely, as multiple items have been tested, but it seems rather unlikely. Some of the ICD-10 coded diagnoses, such as depression or sleep disorder, were rarely recorded and, therefore, may not have enough power to determine statistical differences.

A strength of this study is the prospective study design with the inclusion at a university tertiary pain center of patients with chronic pain and high impairment of their quality of life. Contributing to the strength are the variety of several potentially important psychosocial, socioeconomic, and somatic factors and a broad analysis of the subject.

## 5. Conclusions

In summary, our study again highlights that opioid use is strongly interwoven with a variety of psychological and socioeconomic factors. In addition to the psychological factors of opioid consumption in patients with chronic pain, we found a correlation of opioid use with the occupational status and the IEQ total. Taking occupational status and IEQ into account could be useful for weighting the treatment of pain, e.g., for special psychological, social, and medical support. Therefore, further screening models, e.g., with the help of assessments, could be a requirement for successful multimodal treatment schemes.

## Figures and Tables

**Table 1:** Patient characteristics: Personal data, pain localizations, socioeconomic data, coded diagnoses and scales, and analgesic medications, WSP = widespread pain, IEQ = Injustice Experience Questionnaire, DASS = Depression, Anxiety and Stress Scale, FW7 = Marburg questionnaire on habitual health findings, * during the last 4 weeks, ** total = (current + mean + highest)/3.

		Patients/*n* (%)
Age *		50.3 (SD 14.2)
Sex (m/f)		67/97
Pain localization	Head and Face	30 (18.3%)
	Cervical	23 (14.0%)
	Lumbar	66 (40.3%)
	Extremities	15 (9.1%)
	Abdominal	5 (3.0%)
	WSP	25 (15.2%)
Occupational Status	Retired	19 (11.5%)
	Disability pension	31(18.9%)
	Unemployed	29 (17.6%)
	Employed	85 (51.9%)
Work leave	Yes	58 (35.7%)
	No	66 (40.2%)
	n.a.	40 (24.4%)
Pension application	Yes	12 (7.3%)
	No	123 (75.0%)
	n.a.	29 (17.6%)
Professional education	Academic	27 (16.4%)
	Non-academic	128 (78.0%)
	None	10 (6%)
Marital status	married	100 (60.9%)
	divorced	13 (7.9%)
	widowed	3 (1.8%)
	unwedded	48 (29.3%)
Analgesic medication	Opioids schedule II	16 (9.85)
	Opioids schedule III	26 (15.8%)
	Non-opioids	97 (59.1%)
	Antidepressants	36 (21.9%)
	Anticonvulsants	30 (18.3%)
	Muscle relaxants	5 (3.0%)
	Others	26 (15.8%)
Number of compounds	One compound	91 (55.5%)
	Two compounds	30 (18.3%)
	Three compounds	23 (14.0%)
	>Three compounds	18 (11.0%)
Coded psychological Diagnoses	Patients (*n*)	Patients (*n*)
Pain Disorder with Somatic and Psychological Factors	Yes: 149	No: 15
Depression	Yes: 79	No: 85
Anxiety	Yes: 12	No: 152
Somatization disorder	Yes: 7	No: 157
Sleep disorder	Yes: 83	No: 81
Coded Z-diagnoses		
family	Yes: 52	No: 112
work	Yes: 88	No: 76
biography	Yes: 37	No: 127
finance	Yes: 26	No: 138
any Z-diagnose	Yes: 129	No: 35
Pain scores		Median (IQR)
	Current	7.0 (5.0–8.0)
	Mean *	7.0 (6.0–8.0)
	Highest	9.0 (8.0–10.0)
	Bearable	3.0 (2.0–4.0)
	Total **	7.33 (6.33–8.0)
IEQ	Blame	8.0 (4.0–13.75)
	Severity	15.0 (12.0–18.0)
	Total	24.0 (17.0–31.0)
DASS	Depression	9.0 (4.0–14.0)
	Anxiety	5.0 (2.0–9.0)
	Stress	10.0 (7.0–14.0)
	Total	25.0 (15.0–34.0)
FW 7		10.0 (4.0–14.75)
Disability score		77.33 (56.67–83.33)

**Table 2:** Opioids and Age (years), sex, and different pain localizations, percentages represent within group values, * Mann–Whitney Test, ** Fisher’s exact test, *** chi-squared test, *p* < 0.05 = significant, ^a^ WSP = widespread pain.

		Opioids	No Opioids	*p*
Age *	50.3 (SD 14.2)	56.30 (35.30–67.70)	51.40 (40.30–58.25)	0.1727
Sex (m/f) **	67/97	18/21	49/76	0.4606
Pain *** localization				0.1551
Head and Face		4 (10.3%)	26 (20.8%)	
Cervical		6 (15.4%)	17 (13.6%)	
Lumbar		19 (48.7%)	48 (29.3%)	
Extremities		4 (10.3%)	11 (6.7%)	
Abdominal		3 (7.7%)	2 (1.2%)	
WSP ^a^		3 (7.7%)	21 (12.8%)	

**Table 3:** Opioids and pain scores, * during the last 4 weeks, *p* < 0.05 = significant, Mann–Whitney Test.

Pain Scores	Opioids	No Opioids	
Current	7.0 (5.0–8.0)	6.5 (5.0–8.0)	0.5181
Mean *	8.0 (7.0–9.0)	7.0 (6.0–8.0)	0.0047
Highest	9.0 (8.0–10.0)	9.0 (8.0–10.0)	0.3952
Bearable	3.0 (2.0–4.0)	3.0 (2.0–4.0)	0.4854
Total NRS	7.67 (6.67–8.33)	7.33 (6.33–8.0)	0.2215

**Table 4:** Opioids and psychological factors, Fisher’s exact test, *p*-values = opioids (strong and weak) vs. no opioids.

	Opioids	No Opioids	*p*
IEQ			
IEQ total (all opioids)	26.0 (19.0–33.0)	23.0 (17.0–29.5)	0.1342
IEQ total (only Schedule III opioids)	28.0 (22.5–33.5)	23.0 (17.0–29.5)	*p* = 0.0417
IEQ blame	10.0 (6.0–15.0)	8.00 (4.0–13.0)	0.1270
IEQ severity	16.0 (12.0–19.0)	15.9 (12.0–18.0)	0.2407
DASS			
Depression	13.0 (6.0–18.0)	8.0 (4.0–13.0)	0.0094
Anxiety	6.0 (2.0–11.0)	4.0 (1.0–8.0)	0.0522
Stress	12.0 (8.0–16.0)	10.0 (7.0–14.0)	0.0618
Total	32.0 (17.0–42.0)	22.0 (14.5–33.0)	0.0182
PDI	76.67 (53.33–86.67)	73.33 (56.67–83.33)	0.5097
FW 7	9.0 (3.0–14.0)	10.0 (5.0–15.0)	0.4544
Coded diagnoses			
Pain Disorder with Somatic and Psychological Factors	Yes: 35 No: 4	Yes: 114 No: 11	0.7556
Depression	Yes: 17 No: 22	Yes: 49 No: 76	0.7091
Anxiety	Yes: 3 No: 36	Yes: 9 No: 116	1.0
Sleep disorder	Yes: 22 No: 17	Yes: 61 No: 64	0.4651

**Table 5:** Opioids and social factors ** values missing to 164: n.a., chi-squared test, *p*-values = opioids vs. no opioids.

	All Opioids	No Opioids	*p*
Occupational status			<0.0001
employed	6	71	
unemployed	15	21	
retired	10	9	
disability pension	8	21	
Work leave **	Yes: 12 No: 11	Yes: 44 No: 55	0.6430
Pension application	Yes: 0 No: 28	Yes: 12 No: 95	0.0714
Professional education			0.0994
academic	3	24	
nonacademic	32	96	
none	4	5	
Marital status			0.9862
divorced	3	10	
married	24	76	
unwedded	12	36	
Coded psychosocial diagnoses			
Finance	Yes: 5 No: 34	Yes: 21 No: 104	0.6256
Family	Yes: 9 No: 30	Yes: 43 No: 82	0.2378
Workplace	Yes: 20 No: 19	Yes: 69 No: 56	0.7149
Biography	Yes: 11 No: 28	Yes: 25 No: 100	0.2773

**Table 6:** Logistic regression analysis examining the relation between opioid use (dependent variable) and IEQ total, DASS Depression, mean pain, B: regression coefficient, SE: Standard error.

	B	SE	Wald	df	*p*	Odds Ratio
Regression						
Constant	−0.3441	1.060	10.541	1	0.001	0.032
Age	0.015	0.014	1.020	1	0.313	1.015
Sex	−0.394	0.394	1.002	1	0.317	0.674
IEQ total	−0.016	0.026	0.365	1	0.545	0.984
DASS D	0.094	0.042	4.875	1	0.027	1.098
NRS mean	0.168	0.106	2.508	1	0.113	1.184
Occupation status	−0.146	0.539	0.073	1	0.787	0.864

## Data Availability

The data presented in this study are available on request from the corresponding author. The data are not publicly available for reasons of data protection.
